# Prediction of Oral Cancer Biomarkers by Salivary Proteomics Data

**DOI:** 10.3390/ijms252011120

**Published:** 2024-10-16

**Authors:** Veronica Remori, Manuel Airoldi, Tiziana Alberio, Mauro Fasano, Lorenzo Azzi

**Affiliations:** 1 Department of Science and High Technology, University of Insubria, 22100 Como, Italy; vremori@uninsubria.it (V.R.); tiziana.alberio@uninsubria.it (T.A.); mauro.fasano@uninsubria.it (M.F.); 2 Department of Medicine and Technological Innovation, University of Insubria, 21100 Varese, Italy; manuel.airoldi@uninsubria.it

**Keywords:** OSCC, proteins, salivary biomarkers, differential abundance analysis, PPI network, ORA

## Abstract

Oral cancer, representing 2–4% of all cancer cases, predominantly consists of Oral Squamous Cell Carcinoma (OSCC), which makes up 90% of oral malignancies. Early detection of OSCC is crucial, and identifying specific proteins in saliva as biomarkers could greatly improve early diagnosis. Here, we proposed a strategy to pinpoint candidate biomarkers. Starting from a list of salivary proteins detected in 10 OSCC patients and 20 healthy controls, we combined a univariate approach and a multivariate approach to select candidates. To reduce the number of proteins selected, a Protein–Protein Interaction network was built to consider only connected proteins. Then, an over-representation analysis (ORA) determined the enriched pathways. The network from 172 differentially abundant proteins highlighted 50 physically connected proteins, selecting relevant candidates for targeted experimental validations. Notably, proteins like Heat shock 70 kDa protein 1A/1B, Pyruvate kinase PKM, and Phosphoglycerate kinase 1 were suggested to be differentially regulated in OSCC patients, with implications for oral carcinogenesis and tumor growth. Additionally, the ORA revealed enrichment in immune system, complement, and coagulation pathways, all known to play roles in tumorigenesis and cancer progression. The employed method has successfully identified potential biomarkers for early diagnosis of OSCC using an accessible body fluid.

## 1. Introduction

Oral Squamous Cell Carcinoma (OSCC) accounts for approximately 90% of oral malignancies and is the most representative tumor in the group of Head and Neck Squamous Cell Carcinoma (HNSCC) [1]. With 377,713 new cases recorded in 2020, OSCC represents the 16th most common malignancy worldwide [2]. Risk factors commonly associated with the onset of the disease are tobacco, alcohol, and betel quid consumption, but 10–15% of patients have not been exposed to traditional risks [3]. In addition, there is an alarming increase in the incidence of OSCC in young adults, and this trend is independent of Human Papilloma Virus (HPV) infection, a feature that differentiates this tumor from oropharyngeal cancer [4]. Unfortunately, the most recent advances in the field of cancer therapy, like immunotherapeutic approaches, have not changed the clinical scenario of the disease, with the 5-year survival rate steadily assessed around 50% over the last few decades [5]. Therefore, the only effective strategy to reduce the morbidity and mortality associated with this tumor is early detection, which relies on clinical examination and histopathological assessment of suspected lesions. Within this frame, the detection of surrogate biomarkers in biological fluids associated with the early stages of the disease could represent an innovative auxiliary approach that supports the clinician in adopting an effective prevention strategy [6]. In the case of oral cancer, saliva could represent the biological fluid that is best indicated for the detection of biomarkers, since tumors of the oral cavity are constantly in contact with the secreted saliva for a prolonged period [7].

The interest in salivary proteins has significantly increased in the last five years, leading to the creation of several databases containing data retrieved from untargeted and targeted quantitative proteomics approaches, such as shotgun proteomics and multiple reaction monitoring, respectively. Among them, the Human Salivary Proteome Wiki (HSP Wiki) [8] stores custom-curated experimental data of the saliva proteome. It catalogues all data according to several pieces of information, such as sample ID, sample title, tissue type, institution, disease, and protein count. The HSP Wiki also offers analytic tools to perform differential expression analysis comparing protein levels in selected datasets. The analysis of differential protein abundance is crucial in finding biomarkers that are significantly different between two groups of subjects, namely healthy subjects and disease carriers. The classical technique identifies which proteins are up-regulated and down-regulated through the comparison between the abundance of each protein in two groups [9]. Even if this technique has already been employed in several proteomics works [10,11], the univariate comparison of the protein abundance lacks information on the inter-relationship among proteins, which is a fundamental factor when considering multifactorial diseases, such as OSCC. Thus, it is essential to consider new methods, including the use of multivariate analysis techniques, in conjunction with Network Medicine approaches.

Indeed, in recent years, Network Medicine has been increasingly exploited to obtain global information on the proteins involved in a disease and to identify protein targets for therapy [12]. In this context, Protein–Protein Interaction (PPI) networks are useful to consider the physical interactions (edges) between proteins (nodes) on a genome-wide scale. PPIs are biologically meaningful since many functionally similar proteins form complexes and often carry out their function through interactions with other proteins [13].

PPI networks may be further analyzed in terms of pathways to show how protein clusters are functionally linked to specific biological processes [14]. Pathway analysis is employed to identify activated metabolic or signaling mechanisms from omics data, considering the actual functional factors involved in regulatory pathways [15], and it is usually performed by using an over-representation analysis (ORA). An ORA is a statistical method used to highlight relevant pathways and provide functional explanations for changes in protein abundance revealed by differential quantitative proteomics [16]. ORA determines whether genes from pre-defined datasets are present more than it would be expected in a subset of data. It assumes that relevant pathways can be detected if the number of differentially expressed genes exceeds the number of genes randomly expected within a given pathway or biological process. The probability value for the null hypothesis is typically computed by the Fisher exact test with Benjamini–Hochberg multiple-test correction [17].

Here, using available curated proteomic data, we integrated univariate and multivariate analyses in order to select potential salivary biomarkers for OSCC.

## 2. Results

A dataset comprising 2815 proteins detected in the saliva of 10 OSCC patients and 20 healthy controls was downloaded from the HSP Wiki (Table S1, Figure S1).

**Figure 1:**
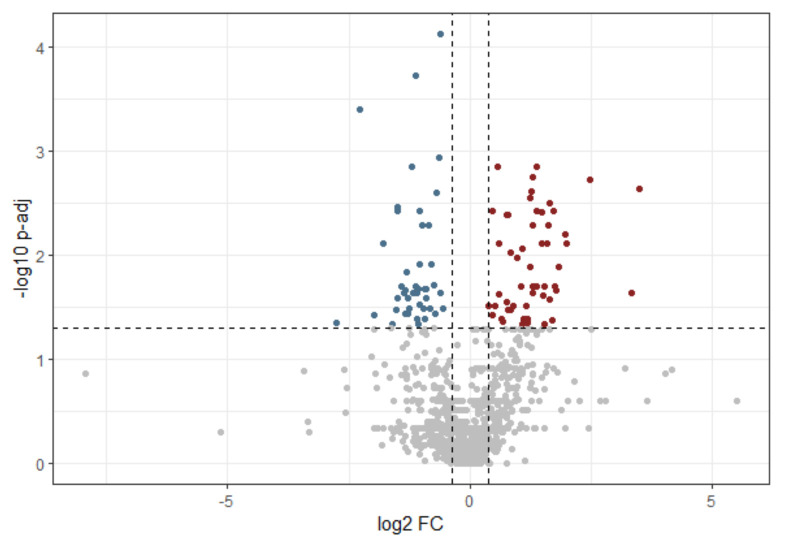
Volcano plot of differential abundance analysis results obtained by using DE analysis tool from HSP Wiki. Red = up-regulated proteins, blue = down-regulated proteins, gray = non-significant differentially abundant proteins (log_2_ (FC) higher than 1.3 or lower than −1.3, representing 30% variation; p-adj > 0.05).

As a first approach, differential abundance analysis was performed, resulting in the identification of 150 differentially abundant proteins with a log_2_ fold change higher than 1.3 or lower than −1.3, representing 30% variation (75 up-regulated, 75 down-regulated) and with an adjusted *p*-value lower than 0.05. This subset of proteins represented around 5.3% of the input list (Figure 1, Table S2).

**Figure 2:**
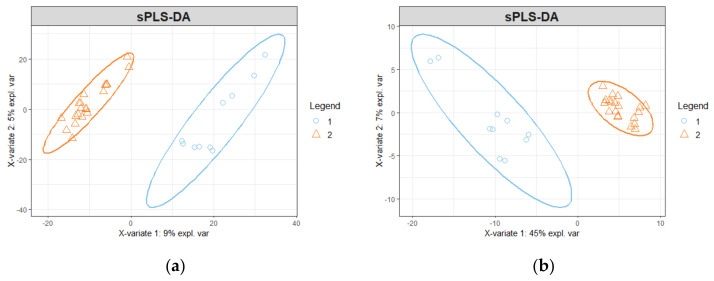
sPLS-DA analysis. Group: 1 = OSCC patients; 2 = healthy controls. (**a**) sPLS-DA performed on initial matrix. X axis = component 1 (it explains 9% of variance). Y axis = component 2 (it explains 5% of variance). (**b**) sPLS-DA performed on final matrix after RFE. X axis = component 1 (it explains 45% of variance). Y axis = component 2 (it explains 7% of variance).

However, the classic univariate approach identifies all those proteins whose level is significantly different between OSCC and healthy controls. Several groups of proteins may change in a correlated way, thus reflecting the presence of protein communities characterized by inter-relationships. To consider such proteins’ inter-relationships, we applied a multivariate approach, based on the Sparse Partial Least Squares Discriminant Analysis (sPLS-DA). After Principal Component Analysis (Figure S2) and the evaluation of the best number of components (Figure S3), the sPLS-DA was performed considering only two components (Figure 2a). The sPLS-DA coupled with recursive feature elimination (RFE) was iteratively applied until 141 proteins were left (5% of the input list). At the end of the process, the first component explained 45% of the variance between the two groups (Figure 2b).

**Figure 3:**
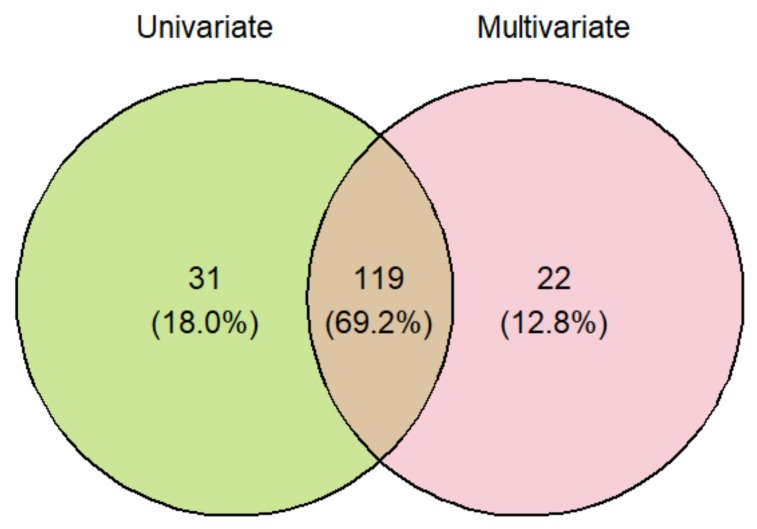
A Venn diagram of the proteoforms selected in the two approaches.

**Figure 4:**
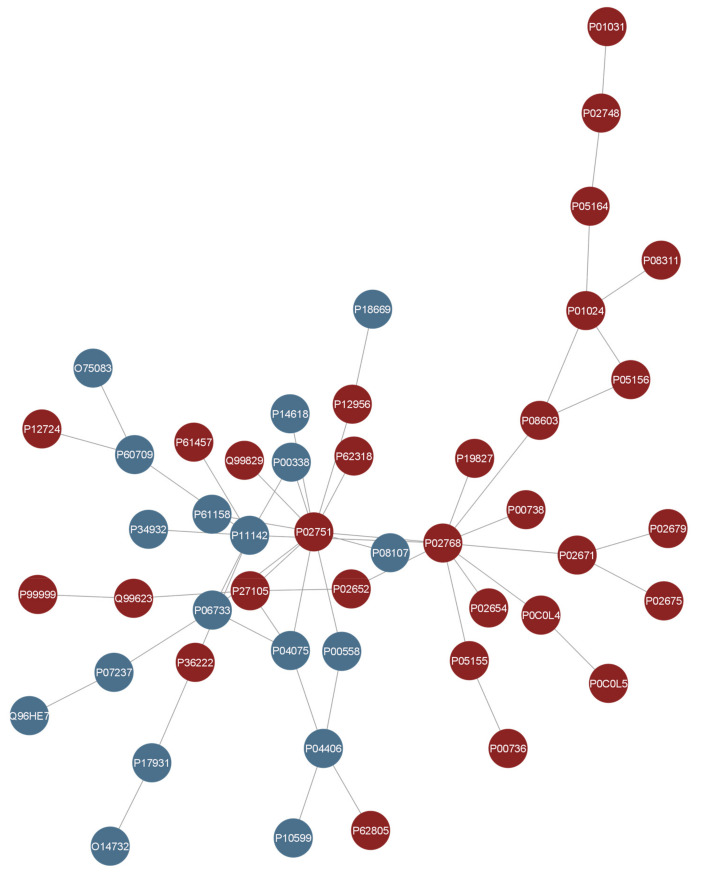
The PPI network formed by 48 nodes and 54 edges. Red = up-regulated proteins and blue = down-regulated proteins according to their log_2_ fold change computed in the univariate approach.

**Figure 5:**
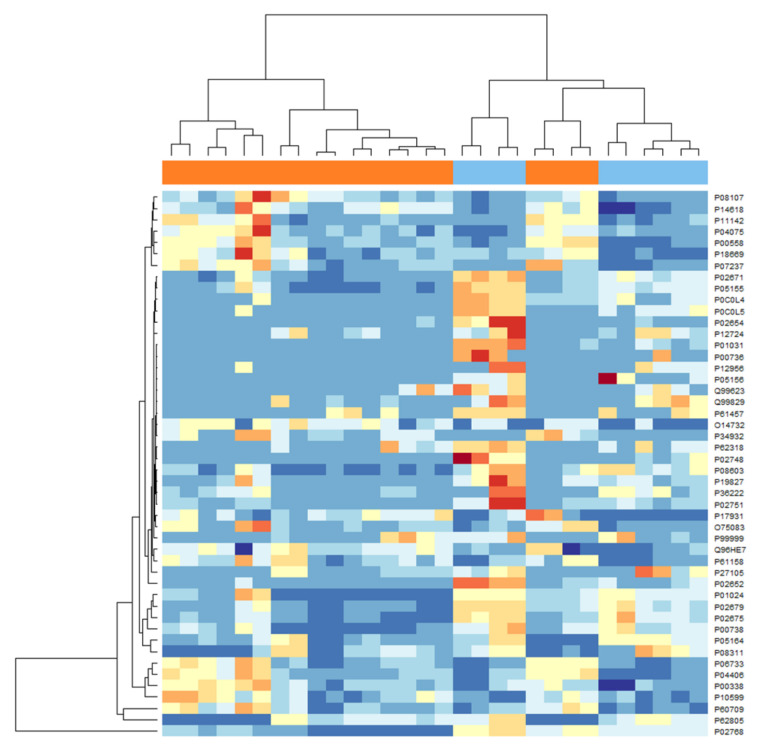
A heatmap of the differential abundance of the 48 connected proteins in the PPI network reported in Figure 4. Colors span from blue (down-regulated) to red (up-regulated). The bar on the top indicates the group: light blue = OSCC patients and orange = healthy controls.

Among the proteoforms selected by the univariate and multivariate approaches, 119 were in common (Figure 3), including 38 isoforms. The combination of the two lists comprised 172 differentially abundant proteoforms. Out of them, 54 were isoforms. Thus, the 118 unique IDs (not considering isoforms) were used as input to query the IMEx database to generate a PPI network. After removing the first interactors, self-loops, and duplicated edges, the resulting PPI network was composed of 48 connected proteins (30 up-regulated, 18 down-regulated) (Figure 4 and Figure 5). These proteins are listed in Table 1. Thus, the PPI network shows proteins directly interacting together among the biomarkers list and were those selected for the further analysis.

**Figure 6:**
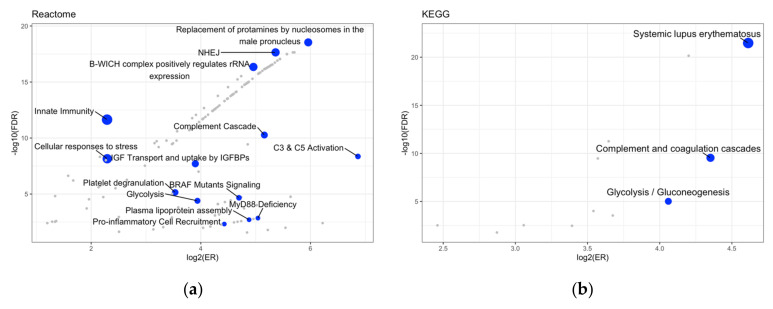
Volcano plots of the ORA results. The size of each dot is related to the number of proteins present in the overlap between the input data and the signature. Blue = pathways selected through affinity propagation. Gray = other enriched pathways. (**a**) Enriched pathways in the Reactome database. (**b**) Enriched pathways in the KEGG database.

**Figure 7:**
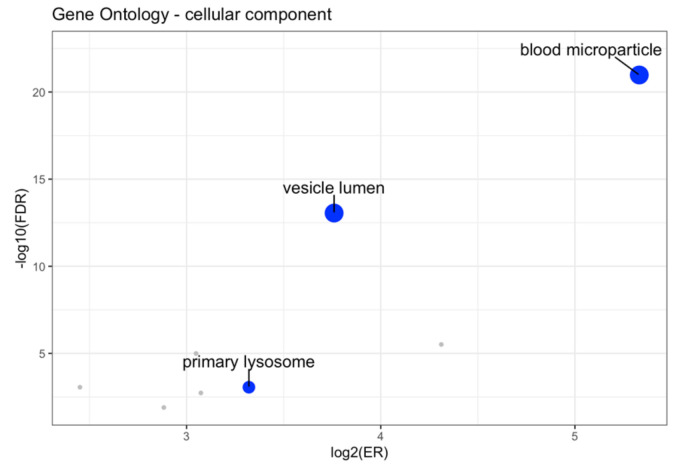
A volcano plot of the ORA results in the Gene Ontology cellular component database. The size of each dot is related to the number of proteins present in the overlap between the input data and the signature. Blue = the GO terms selected through affinity propagation. Gray = other enriched GO terms.

To investigate the functional roles of the mapped proteins within known pathways, we performed an over-representation analysis interrogating KEGG and Reactome as pathway databases, using the 48 proteins listed in Table 1 as the input list. KEGG analysis revealed 12 enriched categories (three after redundancy reduction), while Reactome revealed 156 enriched categories (16 after redundancy reduction). Both analyses showed enrichment in pathways related to immune processes (innate immunity, neutrophil degranulation, complement, and coagulation) among those with a lower FDR (Figure 6). GO cellular component analysis revealed eight enriched categories (three after redundancy reduction), with significant enrichment of proteins localized in the extracellular region (Figure 7). These results showed additional significantly enriched pathways with respect to the ORA performed on the 119 proteoforms resulting from the intersection of the univariate and multivariate analyses (Figure S4).

## 3. Discussion

Oral Squamous Cell Carcinoma represents the most common malignant tumor of the oral cavity, accounting for 90% of oral malignancies. Despite traditional risk factors like tobacco and alcohol consumption, a significant number of patients remain unexposed to these factors. The increasing incidence of OSCC among young adults, regardless of HPV infection, presents a challenge to current therapeutic approaches, with a stagnant 5-year survival rate of approximately 50%. Early detection through biomarker identification in saliva offers a promising avenue for effective prevention strategies against this disease.

In recent years, there has been a surge of interest in salivary proteins, leading to the development of multiple databases. The examination of differential protein abundance plays a crucial role in identifying biomarkers that show significant differences between healthy individuals and those with the disease. By comparing protein abundance levels between these two groups, traditional techniques can identify up-regulated or down-regulated proteins, aiding in the search for effective biomarkers or evidencing proteins involved in the pathogenesis.

However, when studying OSCC, it is imperative to move beyond traditional univariate analysis, which solely compares individual protein abundance levels between OSCC patients and healthy controls. While this approach provides valuable insights into specific proteins, it overlooks the interconnectedness and collective impact of these proteins within the biological system. To gain a more comprehensive understanding of the molecular landscape and how these proteins collectively contribute to OSCC pathogenesis, a multivariate approach is essential.

Thus, the integration of univariate and multivariate approaches is valuable for identifying proteins that not only show significant differences in abundance between the two groups, but also play a crucial role in discriminating between them and evidencing disease molecular cues. The PPI network obtained from the list of 172 differentially abundant proteoforms highlighted 48 proteins as physically connected. This workflow allowed us to select features probably more relevant to the disease and to obtain a list of candidate markers for experimental validation. Indeed, retaining a smaller protein subset could be useful for performing targeted analyses.

Furthermore, the list contained proteins that were already suggested to be differentially regulated in OSCC patients. For instance, starting from the top of the list, the protein with the lowest FDR value was the Heat Shock 70 kDa protein 1A/1B (P08107). Several studies demonstrated that it provides a crucial contribution to oral carcinogenesis, and its expression could potentially be used as predictor of progression and recurrence of OSCC [18]. Also, its down-regulation in cancer cell leads to phenotypic alterations [19]. Additionally, the level of two key glycolytic enzymes, Pyruvate kinase PKM (P14618) and Phosphoglycerate kinase 1 (P00558), was also found to be altered in saliva. These proteins were related to carcinogenesis and OSCC beyond their role in glycolysis [20,21]. Moreover, they contribute to cancer progression and angiogenesis. In addition, the fragments containing Complement C4-B (P0C0L5) can be detected in oral primary tumors and are increased in saliva from patients with OSCC [22]. This protein is one of the chemical barrier proteins suggested to show altered expression between patients with OSCC and controls, along with Fibrinogen alpha chain (P02671), Complement component C9 (P02748), Complement factor I (P05156), Fibrinogen beta chain (P02675), Complement C5 (P01031), Fibrinogen gamma chain (P02679), Plasma protease C1 inhibitor (P05155), Complement C3 (P01024), Complement factor H (P08603), Apolipoprotein A-II (P02652), Eosinophil cationic protein (P12724), Inter-alpha-trypsin inhibitor heavy chain H1 (P19827), Haptoglobin (P00738), and Complement C1r subcomponent (P00736). All of these proteins were more present in the saliva of patients with OSCC compared with controls [23].

To the best of our knowledge, the other proteins listed in Table 1 have not yet been associated with OSCC and may be considered new potential biomarkers that require further validation.

Finally, to understand the biological significance of the identified proteins, an ORA was performed. The 48 connected and differentially expressed proteins were enriched in pathways related to immune system, complement, and coagulation processes. Alterations in immune system processes are known to enhance tumorigenesis and cancer progression [24]. Furthermore, complement processes (as part of immune processes) have a role in the initiation of OSCC and other tumor types [25,26]. Coagulation is the process by which a blood clot is generated. OSCC and other cancer types are frequently associated with hemostatic derangement [27,28]. However, given that these pathways are very generic, the specificity of our signature should be verified. For instance, it would be crucial to distinguish between minor oral lesions and actual tumor lesions. To this purpose, it would be useful to collect more data by expanding the sample size, especially of the control group, in order to enhance the specificity and the diagnostic utility of this signature.

As expected, since our data are related to saliva, Gene Ontology analysis demonstrated an enrichment in proteins localized to the extracellular region. Worthy of note is that the most significant term is “blood microparticles”. Microparticles are phospholipid microvesicles derived from different cell types, e.g., blood cells and platelets, recently related to coagulation and angiogenesis in OSCC patients [29].

In conclusion, the combined univariate and multivariate approach, along with the construction of a Protein–Protein Interaction network, has proven to be a robust and effective method for identifying potential biomarkers in OSCC. By narrowing down the list of proteins to those physically connected in the network, the analysis has provided a focused set of candidates for further experimental validation. To this end, in the near future, we plan to analyze a homogeneous cohort of independently enrolled patients to assess the sensitivity and specificity of our proposed signature. This will be achieved using machine learning approaches, such as the Sparse Partial Least Squares Discriminant Analysis with a training set and a test set for cross validation [30,31].

Moreover, the over-representation analysis further strengthened the validity of the findings by revealing enrichment in key pathways related to tumorigenesis and cancer progression. This method not only offers insights into the molecular mechanisms underlying oral cancer but also holds promise for improving early diagnosis and personalized treatment strategies in the future. Indeed, new signatures specific for treatment response could be obtained by enrolling new subjects stratified in terms of treatment response.

## 4. Materials and Methods

### 4.1. Differential Abundance Analysis

Lists of proteins detected in saliva in 10 OSCC patients and 20 healthy subjects were downloaded from the HSP Wiki, selecting samples provided by the J. Craig Venter Institute. The inclusion criteria for their study required that patients had undergone surgery as their primary treatment, while those who had received prior chemotherapy, radiation, or other cancer medications were excluded [32].

To retrieve more information, two different approaches were adopted to analyze the differential protein abundance: a univariate approach and a multivariate approach. Using the DE analysis tool from the HSP Wiki, the expression in the two groups (OSCC and healthy) was compared for each protein by computing the log_2_ mean fold changes and employing the Wilcoxon rank-sum test. The fold change value was computed as the ratio between the average abundance of the two groups (OSCC/healthy). The resulting *p*-values were corrected to the false discovery rate (FDR) according to the Benjamini–Hochberg procedure. Only proteins with an expression difference higher than ±30% (up-regulated or down-regulated) and with an adjusted *p*-value lower than 0.05 were selected.

Then, we developed a novel method using a script in R (version 4.2.0.) to identify proteins that significantly influenced the distinction between the two groups (OSCC and healthy) using the Sparse Partial Least Squares Discriminant Analysis (sPLS-DA) [31]. This classifier is a variation of the sPLS regressor [33]. In essence, the algorithm maximized the distance between scores assigned to individuals grouped by a specific factor. The scores ($S_{j}$) were derived through a unitary transformation of the covariance matrix, ensuring that the elements were not part of a single eigenstructure. The diagonalization of such matrix led to the transformation matrix U and the diagonal matrix D so that U×Z=D×Z, where Z is the penalized covariance matrix. Therefore, U contained the eigenvectors $\underset{u_{i}}{\to }$ and D the eigenvalues $d_{\mathrm{ii}}$. Each sample j could be represented in a low-dimensionality space with one or two scores that were computed as follows:

$$S_{j}=\sum_{i}u_{i1}r_{\mathrm{ij}}$$

where $r_{\mathrm{ij}}$ indicates the abundance of protein i in sample j. The $u_{i1}$ values are the loadings on the first component, i.e., the vector with the weights of the contribution of each original variable to the corresponding latent component. A recursive feature elimination (RFE) technique was incorporated into the sPLS-DA to eliminate proteins with less impact on the discrimination between the two groups, selecting the protein with the lower loading $u_{i1}$. The sPLS-DA coupled with RFE was iteratively applied until 141 proteins were left (5% of the input list).

Finally, the two lists of proteins obtained from the two approaches were merged to consider proteins that are significantly differentially abundant and that also contribute the most to the difference between the two groups.

### 4.2. PPI Network Generation Workflow

Cytoscape 3.9.1 was used to generate a PPI network starting from the selected proteins to investigate their physical interactions [34]. The public database of the International Molecular Exchange Consortium (IMEx) was queried through Cytoscape using the PSICQUIC standard (the Proteomics Standard Initiative Common QUery InterfaCe) created by the Human Proteome Organization Proteomics Standards Initiative (HUPO-PSI) in 2008 [35]. First, the list of proteins was used as an input list to generate a PPI network encompassing all of their first interactors. The network was filtered for interactor type and for taxonomy ID 9606 (*Homo sapiens*) to remove homology inferences. Then, the first interactors were removed to keep only interactions between the input proteins. Also, duplicated edges and self-loops were removed. Only connected proteins were considered under the assumption that interacting proteins may participate in the same biochemical process at a certain time.

### 4.3. Over-Representation Analysis

An over-representation analysis was performed utilizing Webgestalt, a web application designed for enrichment analysis [36]. Webgestalt enables users to extract biological information from a list of genes (or proteins) derived from omics studies. The ORA was performed using the list of proteins retrieved from our analysis as input, setting *Homo sapiens* as the organism of interest. As functional databases, Kyoto Encyclopedia of Genes and Genomes (KEGG) [37] and Reactome [38] were selected as pathway databases. Furthermore, the Gene Ontology cellular component database [39] was incorporated. As a genomic landscape for statistical testing, “genome protein coding” was selected. Enriched categories with a Benjamini–Hochberg false discovery rate (FDR) ≤ 0.05 were considered statistically significant. To remove redundancy in the output of the ORA, the affinity propagation algorithm was employed, and the remaining pathways were selected.

## Figures and Tables

**Table 1:** The list of connected proteins annotated with the log_2_ FC value and the FDR of the univariate analysis ^a^.

Uniprot Accession	Protein Name	log_2_ FC	FDR
P08107	Heat shock 70 kDa protein 1A/1B	−0.62	0.000075
P14618	Pyruvate kinase PKM	−1.12	0.000187
P00558	Phosphoglycerate kinase 1	−2.28	0.000396
P0C0L5	Complement C4-B	2.48	0.001869
P07237	Protein disulfide-isomerase	−0.68	0.002520
P02671	Fibrinogen alpha chain	1.73	0.003736
P02748	Complement component C9	1.39	0.003736
P05156	Complement factor I	0.47	0.003736
P04406	Glyceraldehyde-3-phosphate dehydrogenase	−1.50	0.003736
P02675	Fibrinogen beta chain	1.48	0.003894
P02751	Fibronectin	1.63	0.005073
P01031	Complement C5	0.59	0.007604
P02679	Fibrinogen gamma chain	1.60	0.007648
P05155	Plasma protease C1 inhibitor	1.49	0.007648
P01024	Complement C3	1.08	0.008591
P08603	Complement factor H	0.97	0.010488
P10599	Thioredoxin	−1.02	0.012139
P05164	Myeloperoxidase	1.23	0.012872
P34932	Heat shock 70 kDa protein 4	−0.73	0.019327
P02768	Serum albumin	1.05	0.019750
P62805	Histone H4	1.38	0.020198
P18669	Phosphoglycerate mutase 1	−1.07	0.021136
P04075	Fructose-bisphosphate aldolase A	−0.89	0.021136
P02652	Apolipoprotein A-II	3.34	0.023220
P60709	Actin, cytoplasmic 1	−0.60	0.023220
Q96HE7	ERO1-like protein alpha	−0.90	0.025640
P06733	Alpha-enolase	−1.04	0.029925
P12956	X-ray repair cross-complementing protein 6	0.40	0.030974
P11142	Heat shock cognate 71 kDa protein	−0.56	0.032450
P00338	L-lactate dehydrogenase A chain	−0.83	0.032450
Q99829	Copine-1	0.85	0.032993
P17931	Galectin-3	−1.32	0.036387
P61158	Actin-related protein 3	−1.28	0.036525
Q99623	Prohibitin-2	0.66	0.040969
P99999	Cytochrome c	1.19	0.040969
O75083	WD repeat-containing protein 1	−1.08	0.040969
P12724	Eosinophil cationic protein	1.69	0.042247
P19827	Inter-alpha-trypsin inhibitor heavy chain H1	0.69	0.043209
P00738	Haptoglobin	1.18	0.044182
P62318	Small nuclear ribonucleoprotein Sm D3	1.09	0.045532
P61457	Pterin-4-alpha-carbinolamine dehydratase	0.87	0.050922
P02654	Apolipoprotein C-I	2.52	0.051262
P00736	Complement C1r subcomponent	0.33	0.051262
P08311	Cathepsin G	1.38	0.053989
O14732	Inositol monophosphatase 2	−0.43	0.101858
P0C0L4	Complement C4-A	1.60	0.115126
P36222	Chitinase-3-like protein 1	1.09	0.125089
P27105	Erythrocyte band 7 integral membrane protein	1.50	0.150589

^a^ Proteins with an FDR > 0.05 are those added by the multivariate approach.

## Data Availability

The data presented in this study were derived from the following resources available in the public domain: https://www.salivaryproteome.org/salivary-protein (accessed on 30 July 2024).

## Supplementary Materials

The following supporting information can be downloaded at: https://www.mdpi.com/article/10.3390/ijms252011120/s1.
