# Supplementary material for: Prediction of Oral Cancer Biomarkers by Salivary Proteomics Data
Source: Int J Mol Sci. 2024 Oct 16;25(20):11120. doi: 10.3390/ijms252011120 (PMC11508456; doi:10.3390/ijms252011120)
Supplement: Supplementary file 1 [file ijms-25-11120-s001.zip › Supplementary Appendix_Correct.pdf]

SUPPLEMENTARY FIGURES

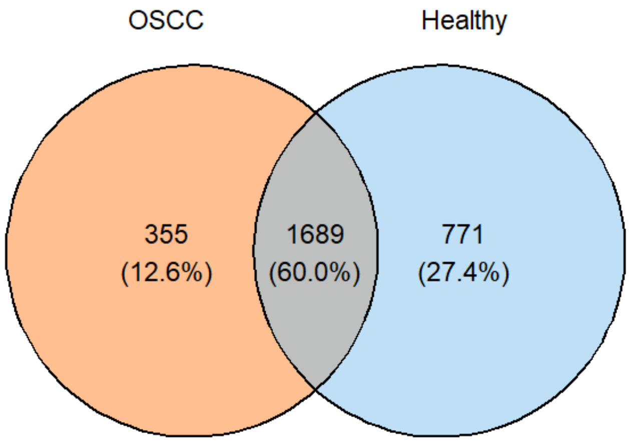

Figure S1. Venn diagram of the input list of 2,815 proteins detected in the saliva of 10 OSCC patients and of 20 healthy controls downloaded from the HSP Wiki.

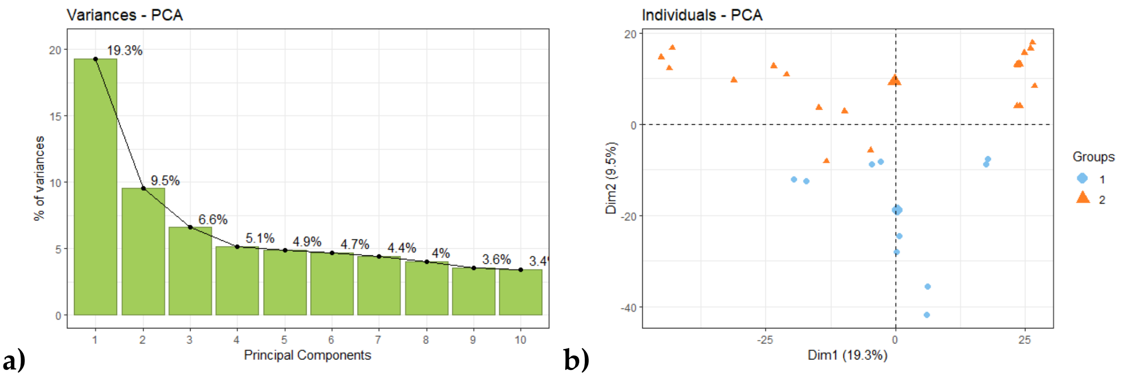

Figure S2. PCA analysis of the whole dataset. a) Percentage of variances explained by each principal component. b) Individuals with a similar profile grouped together.

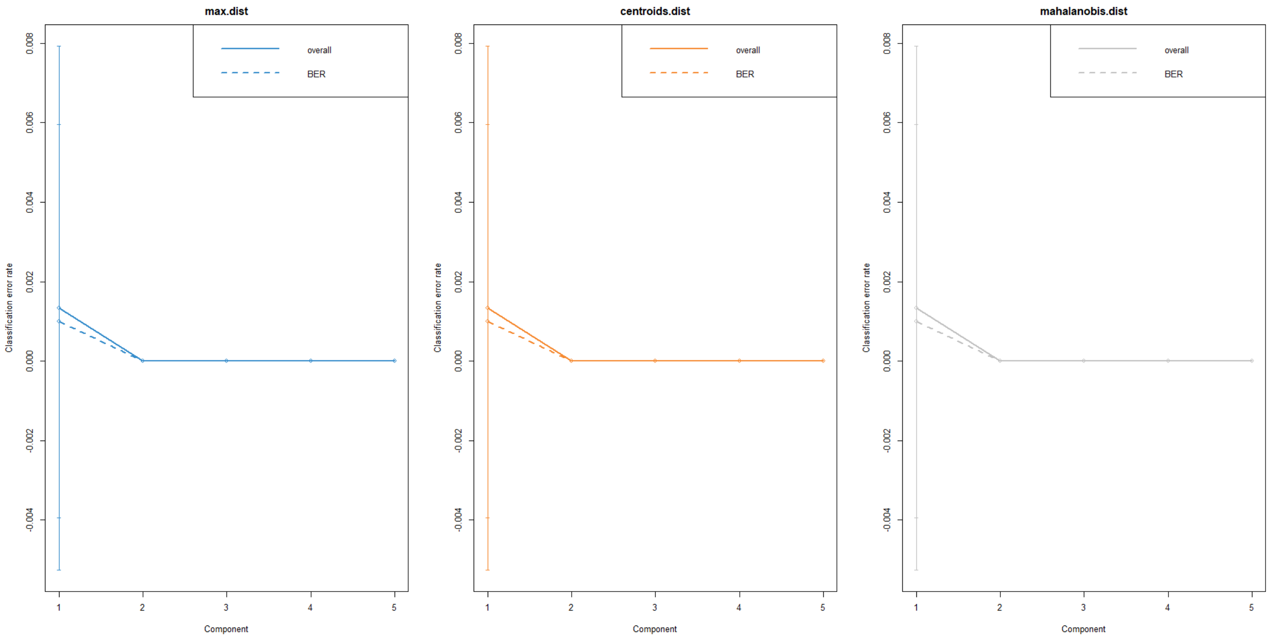

Figure S3. Classification error rate by means of M-fold cross validation. The error rate is averaged across 10 folds repeated 50 times for all prediction distances. BER indicates the balanced error rate, which accounts for unbalanced number of samples per class.

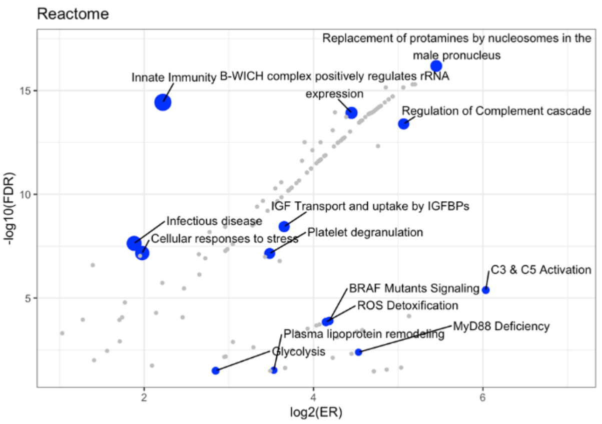

(a)

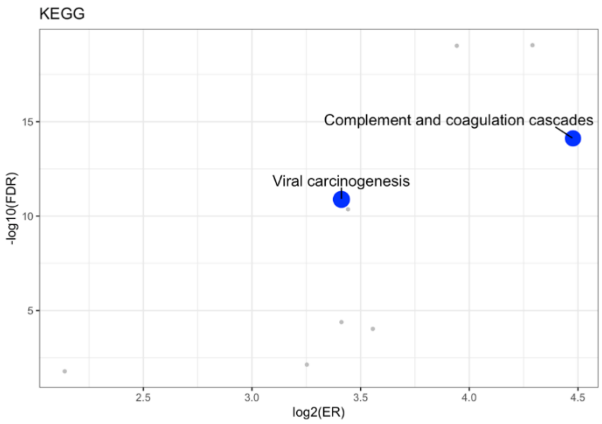

(b)

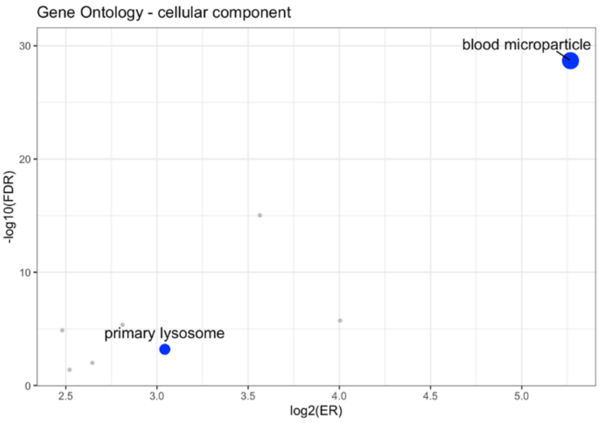

(c)

Figure S4. Volcano plots of the ORA results starting from the list of 119 proteoforms selected by the intersection of the two approaches. The size is related to the number of proteins present in the overlap between the input data and the signature. Blue = pathway selected through affinity propagation. Gray = other enriched pathways. (**a**) Enriched pathways in the Reactome database. (**b**) Enriched pathways in the KEGG database. **(c)** Enriched pathways in the Gene Ontology, Cellular Component database.
